# Lack of practical identifiability may hamper reliable predictions in COVID-19 epidemic models

**DOI:** 10.1126/sciadv.abg5234

**Published:** 2022-01-19

**Authors:** Luca Gallo, Mattia Frasca, Vito Latora, Giovanni Russo

**Affiliations:** 1 Department of Physics and Astronomy, University of Catania, Catania 95125, Italy.; 2 INFN Sezione di Catania, Via S. Sofia, 64, Catania 95125, Italy.; 3 Department of Electrical, Electronics and Computer Science Engineering, University of Catania, Catania 95125, Italy.; 4 Istituto di Analisi dei Sistemi ed Informatica “A. Ruberti,” Consiglio Nazionale delle Ricerche (IASI-CNR), 00185 Roma 00185, Italy.; 5 School of Mathematical Sciences, Queen Mary University of London, London E1 4NS, UK; 6 Complexity Science Hub Vienna, A-1080 Vienna, Austria.; 7 Department of Mathematics and Computer Science, University of Catania, Catania 95125, Italy.

## Abstract

Compartmental models are widely adopted to describe and predict the spreading of infectious diseases. The unknown parameters of these models need to be estimated from the data. Furthermore, when some of the model variables are not empirically accessible, as in the case of asymptomatic carriers of coronavirus disease 2019 (COVID-19), they have to be obtained as an outcome of the model. Here, we introduce a framework to quantify how the uncertainty in the data affects the determination of the parameters and the evolution of the unmeasured variables of a given model. We illustrate how the method is able to characterize different regimes of identifiability, even in models with few compartments. Last, we discuss how the lack of identifiability in a realistic model for COVID-19 may prevent reliable predictions of the epidemic dynamics.

## INTRODUCTION

The pandemic caused by severe acute respiratory syndrome coronavirus-2 is challenging humanity in an unprecedented way (*1*), with the disease, which in a few months has spread around the world, affecting large parts of the population (*2*, *3*) and often requiring hospitalization or even intensive care (*4*, *5*). Mitigating the impact of coronavirus disease 2019 (COVID-19) urges synergistic efforts to understand, predict, and control the many, often elusive, facets of the complex phenomenon of the spreading of a previously unknown virus, from RNA sequencing to the study of the virus pathogenicity and transmissibility (*6*, *7*) to the definition of suitable epidemic spreading models (*8*) and the investigation of nonpharmaceutical intervention policies and containment measures (*9*–*12*). In particular, a large number of epidemic models have recently been proposed to describe the evolution of COVID-19 and evaluate the effectiveness of different counteracting measures, including social distancing, testing, and contact tracing (*13*–*19*). However, even the adoption of well-consolidated modeling techniques, such as the use of mechanistic models at the population level based on compartments, poses fundamental problems. First of all, the very same choice of the dynamical variables to use in a compartmental model is crucial; as such, variables should adequately capture the spreading mechanisms and need to be tailored to the specific disease. This step is not straightforward, especially when the spreading mechanisms of the disease are still unknown or only partially identified. In addition, some of the variables considered might be difficult to measure and track as, for instance, in the case of COVID-19, it occurred in the number of individuals showing mild or no symptoms. Second, compartmental models, usually, involve a number of parameters, including the initial values of the unmeasured variables, which are not known and need to be estimated from data.

Having at disposal a large amount of data, unfortunately, does not simplify the problem of parameter estimation and prediction of unmeasured states. Once a model is formulated, it may occur that some of its unknown parameters are intrinsically impossible to determine from the measured variables or that they are numerically very sensitive to the measurements themselves. In the first case, it is the very same structure of the model to hamper parameter estimation as the system admits infinitely many sets of parameters that fit the data equally well; for this reason, this problem is referred to as structural identifiability (*20*, *21*). In the second case, although under ideal conditions (i.e., noise-free data and error-free models) the problem of parameter estimation can be uniquely solved for some trajectories, it may be numerically ill conditioned, such that from a practical point of view, the parameters cannot be determined with precision even if the model is structurally identifiable (*22*). This situation typically occurs when large changes in the parameters entail a small variation of the measured variables, such that two similar trajectories may correspond to very different parameters (*23*). The term practical identifiability is adopted in this case.

Identifiability in general represents an important property of a dynamical system, as in a nonidentifiable system, different sets of parameters can produce the same or very similar fits of the data. Consequently, predictions from a nonidentifiable system become unreliable. In the context of epidemics forecasting, this means that even if the model considered is able to reproduce the measured variables, a large uncertainty may affect the estimated values of the parameters and the predicted evolution of the unmeasured variables (*24*).

The problem of practical identifiability of model parameters has been investigated using different methodologies based on Fisher’s information theory (*25*, *26*), profile likelihood (*27*), Monte Carlo simulations (*28*), and other computational approaches (*29*). However, the lack of practical identifiability can also affect the reliability of the prediction of the unmeasured variables dynamics (*27*), an issue of utmost importance in the context of COVID-19, which nevertheless still requires a systematic investigation. In particular, an approach to simultaneously characterize the problem of sensitivity to parameters and that of the reliability of predictions of unmeasured variables is still missing.

In more detail, in this paper, we investigate the problem of the practical identifiability of dynamical systems whose state includes not only measurable but also hidden variables, as is the case of compartment models for COVID-19 epidemic. We present a general framework to quantify not only the sensitivity of the measured variables of a given model on its parameters but also the sensitivity of the unmeasured variables on the parameters and on the measured variables. This will allow us to introduce the notion of practical identifiability of the hidden variables of a model. As a relevant and timely application, we show the variety of different regimes and levels of identifiability that can appear in epidemic models, even in the simplest case of a four compartment system. Last, we study the actual effects of the lack of practical identifiability in more sophisticated models introduced for COVID-19.

## RESULTS

### Dynamical systems with hidden variables

Consider the *n*-dimensional dynamical system described by the following equations

$$\begin{array}{c} \overset{\cdot }{m}=f(m,h,q),\\ \overset{\cdot }{h}=g(m,h,q) \end{array}$$ (1)

where we have partitioned the state variables into two sets: the variables **m** ∈ ℝ*^n_m_^* that can be empirically accessed (measurable variables) and those **h** ∈ ℝ*^n_h_^*, with *n_m_* + *n_h_* = *n*, that cannot be measured (hidden).

The dynamics of the system is governed by the two Lipschitz-continuous functions **f** and **g**, which also depend on a vector of structural parameters **q** ∈ Ω*_q_* ⊂ ℝ*^n_q_^*. The trajectories **m**(*t*) and **h**(*t*) of system in eq. 1 are uniquely determined by the structural parameters **q** and by the initial conditions **m**(0) = **m_0_**, **h**(0) = **h_0_**. Here, we assume that some of the quantities **q** are known, while the others are not known and need to be determined by fitting the trajectories of measurable variables **m**(*t*).

We denote by **p** ∈ Ω*_p_* ⊂ ℝ*^n_p_^*, the set of unknown parameters that identify the trajectories, which comprises the unknown terms of **q** and the unknown initial conditions ***h*_0_**. The initial values of the hidden variables are not known and act indeed as parameters for the trajectories generated by system in eq. 1. The initial conditions of the measurable variables **m_0_** may be considered fitting parameters as well.

System in eq. 1 is said to be structurally identifiable when the measured variables satisfy (*21*)

$$m(t,\hat{p})=m(t,p),\forall t\geq 0\Rightarrow \hat{p}=p$$ (2)

for almost any **p** ∈ Ω*_p_*. Notice that, as a consequence of the existence and uniqueness theorem for the initial value problem, if system in eq. 1 is structurally identifiable, the hidden variables can also be uniquely determined.

Structural identifiability guarantees that two different sets of parameters do not lead to the same time course for the measured variables. When this condition is not met, one cannot uniquely associate a data fit to a specific set of parameters or, equivalently, recover the parameters from the measured variables (*23*).

### Assessing the practical identifiability of a model

Structural identifiability, however, is a necessary but not sufficient condition for parameters estimation, so that when it comes to use a dynamical system as a model of a real phenomenon, it is fundamental to quantify the practical identifiability of the dynamical system.

To do this, we consider a solution, $\bar{m}(t)=m(t,\bar{p})$ and $\bar{h}(t)=h(t,\bar{p})$, obtained from parameters $p=\bar{p}$, and we explore how much the functions **m**(*t*) and **h**(*t*) change as we vary the parameters $\bar{p}$ by a small amount **δ*p***. To first order approximation in the perturbation of the parameters, we have $\delta m=\frac{\partial m}{\partial p}\delta p+O(∥\delta p{∥}^{2})$ and $\delta h=\frac{\partial h}{\partial p}\delta p+O(∥\delta p{∥}^{2})$.

Hence, by dropping the higher order terms, we have $∥\delta m{∥}^{2}=\int_{0}^{\infty }{|\delta m|}^{2}\text{dt}=\delta p^{T}M\delta p$ and $∥\delta h{∥}^{2}=\int_{0}^{\infty }{|\delta h|}^{2}\text{dt}={\delta p}^{T}H\delta p$, where the entries of the sensitivity matrices $M=M(\bar{p})\in {\mathbb{R}}^{n_{p}\times n_{p}}$ and $H=H(\bar{p})\in {\mathbb{R}}^{n_{p}\times n_{p}}$ for the measured and unmeasured variables are defined as

$${\left(M\right)}_{\text{ij}}=\int_{0}^{\infty }\frac{\partial m^{T}}{\partial p_{i}}\frac{\partial m}{\partial p_{j}}\text{dt};\;{\left(H\right)}_{\mathrm{ij}}=\int_{0}^{\infty }\frac{\partial h^{T}}{\partial p_{i}}\frac{\partial h}{\partial p_{j}}\text{dt}$$ (3)

Note that these matrices are positive semidefinite by construction. The smallest change in the measured variables **m**(*t*) will take place if **δp** is aligned along the eigenvector **v**_1_ of M corresponding to the smallest eigenvalue λ_1_(M). Hence, we can consider $\sigma =\sqrt{\lambda_{1}(M)}$ to quantify the sensitivity of the measured variables to the parameters. Practical identifiability requires high values of σ as these indicate cases where small changes in the parameters may produce considerable variations of the measurable variables, and therefore, the estimation of the model parameters from fitting is more reliable.

Suppose now we consider a perturbation, **δp**_1_, of the parameters aligned along the direction of **v_1_**. We can evaluate the change in **h**(*t*) due to this perturbation by

$$\eta^{2}=\frac{\delta p_{1}^{T}H\delta p_{1}}{\delta p_{1}^{T}\delta p_{1}}$$ (4)

The value of η quantifies the sensitivity of the hidden variables to the parameters of the model, when these parameters are estimated from the fitting of the observed variables since ∥**δh** ∥ = η ∥ **δp**_1_∥.

Notice that in this case, and differently from σ, lower values of η are desirable because they imply a better prediction on the hidden variables.

Last, with the help of the sensitivity matrices defined above, we can also evaluate the sensitivity of the hidden variables to the measured variables as

$$\mu^{2}=\underset{∥\delta p∥=1}{\text{max}}\frac{\delta p^{T}H\delta p}{\delta p^{T}M\delta p}$$ (5)

This parameter is of particular relevance here, since it provides a bound on how the uncertainty on the measured variables affects the evolution of the hidden variables. In addition, the parameter μ^2^ can be efficiently computed as it corresponds to the maximum generalized eigenvalue of matrices (H, M), as shown in Materials and Methods.

The sensitivity matrices are useful in studying the effect of changing the number of hidden variables and unknown parameters on the practical identifiability of a model. Assume that we have access to one more variable, thus effectively increasing the size of the set of measured variables to *n_m_*′ = *n_m_* + 1 and, correspondingly, reducing that of the unmeasured variables to *n_h_*′ = *n_h_* − 1. This corresponds to considering new variables **m**′ and **h**′. From the definition in Eq. 3, the new sensitivity matrix can be written as M′ = M + M_1_, where M_1_ is the sensitivity matrix for the newly measured variable. Given Weyl’s inequality [page 239 of (*30*)], we have that λ_1_(M^′^) ≥ λ_1_(M) + λ_1_(M_1_) and since M_1_ is also positive semidefinite, λ_1_(M^′^) ≥ λ_1_(M). This means that measuring one further variable (or more than one) of the system increases the practical identifiability of a model, as expected. As H^′^ = H − M_1_, it is also possible to demonstrate that μ(M^′^) ≤ μ(M) (see Materials and Methods). Let us now consider a different scenario: Suppose we have a priori knowledge of one of the model parameters so that we do not need to estimate its value by fitting the model to the data. In this case, we can define new sensitivity matrices $\tilde{M},\tilde{H}\in {\mathbb{R}}^{\left(n_{p}-1)\times (n_{p}-1\right)}$ for the measured and unmeasured variables, respectively. Given the Cauchy’s interlacing theorem [page 242 of (*30*)], we have that $\lambda_{1}(\tilde{M})\geq \lambda_{1}(M)$, which implies that practical identifiability is improved by acquiring a priori information on some of the model parameters. For instance, in the context of COVID-19 models, one may decide to fix some of the parameters, such as the rate of recovery, to values derived from medical and biological knowledge (*24*, *31*–*33*) and to determine from fitting the more elusive parameters, such as the percentage of asymptomatic individuals or the rates of transmission.

The sensitivity measures we have introduced point out that prior knowledge of some of the parameters, or a larger set of measurable variables, reduces the sensitivity of the measured variables to the parameters and that of the hidden variables to a variation in the measured ones. However, gathering further knowledge can be difficult or even not possible so that these results, which should not be interpreted as an oversimplified solution to the problem of identifiability, have to be considered in the light of practical issues that might arise in the measurement of the model variables and parameters.

### The sensitivity measures reveal different regimes of identifiability

**Fig. 1.**
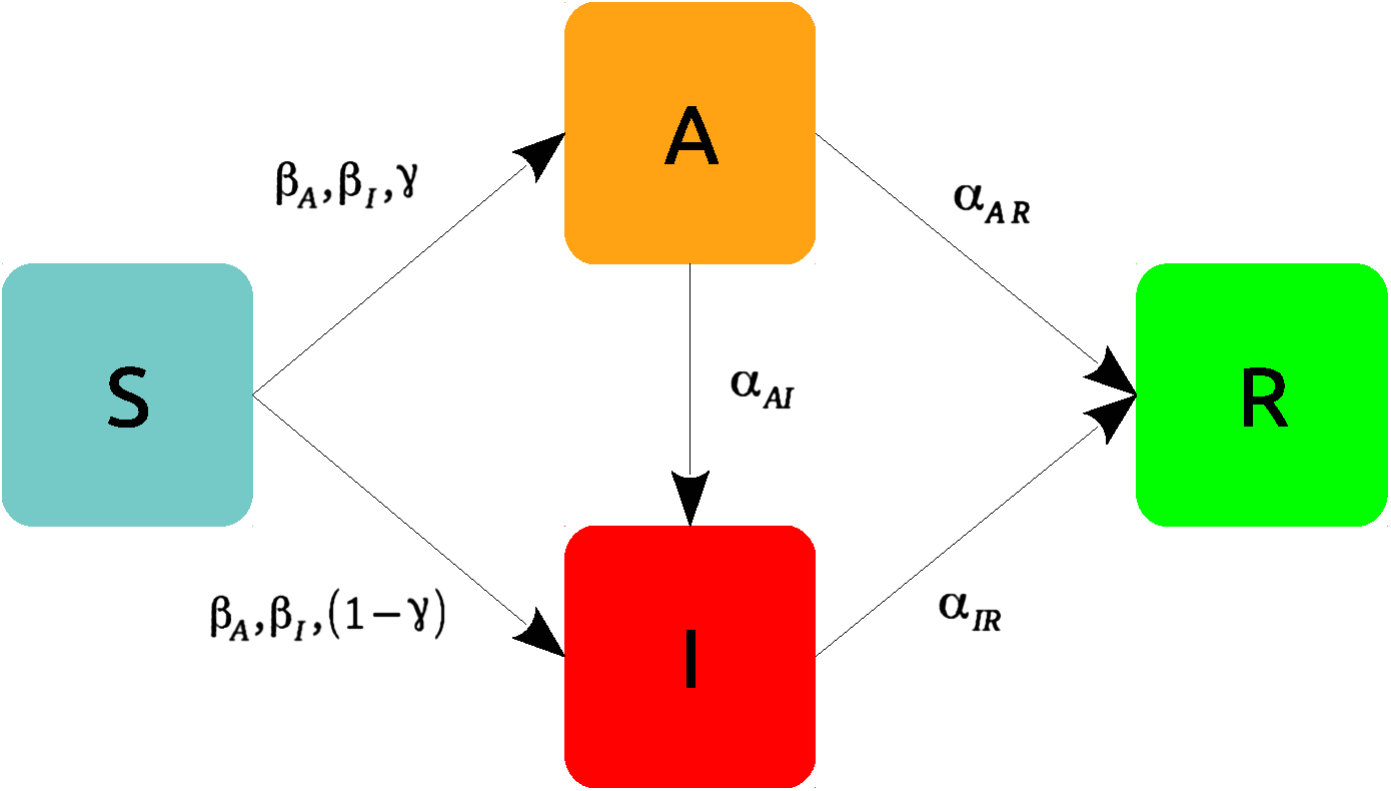
Graphical representation of a SIAR model in which infectious individuals can either be symptomatic (I) or asymptomatic (A) (see also Eq. 21 in Materials and Methods).

As a first application, we study the practical identifiability of a four compartment mean-field epidemic model (*34*) in the class of SIAR models (*35*), developed to assess the impact of asymptomatic carriers of COVID-19 (*8*, *36*, *37*) and other diseases (*38*–*40*). In such a model (Fig. 1), a susceptible individual (*S*) can be infected by an infectious individual who can either be symptomatic (*I*) or asymptomatic (*A*). The newly infected individual can either be symptomatic (*S* → *I*) or asymptomatic (*S* → *A*). Furthermore, we also consider the possibility that asymptomatic individuals develop symptoms (*A* → *I*), thus accounting for the cases in which an individual can infect before and after the onset of the symptoms (*41*). Last, we suppose that individuals cannot be reinfected as they acquire a permanent immunity (*R*).

One of the crucial aspects of COVID-19 is the presence of asymptomatic individuals who are difficult to trace as the individuals themselves could be unaware about their state. Consequently, we assume that the fraction of asymptomatic individuals, *a*(*t*), is not measurable, while the fractions of symptomatic, ι(*t*), and recovered, *r*(*t*), are measured variables, that is, **m** ≡ [ι, *r*] and **h** ≡ [*s*, *a*]. As mentioned above, practical identifiability is a property of the trajectories of the system, which are uniquely determined by the values of the unknown parameters **p**. Here, we illustrate how the sensitivity of both measured and unmeasured variables changes with the probability γ that a newly infected individual shows no symptoms when all the other parameters of the model are fixed (to the values reported in Materials and Methods). Concerning the choice of vector **p**, here, we consider the following case. First, as the number of symptomatic infectious and recovered individuals are supposed to be measurable, we have assumed the initial conditions ι(0), *r*(*0*), and the recovery rate of the symptomatic individuals, i.e., α_IR_,to be known quantities. Second, we assume to be able to measure, for instance, through backward contact tracing, the rate at which asymptomatic individuals develop symptoms, i.e., α_AI_. Hence, the parameters to be determined are the remaining ones, i.e., **p** = [*a*(0), β*_I_*, β*_A_*, γ, α*_AR_*].

**Fig. 2.**
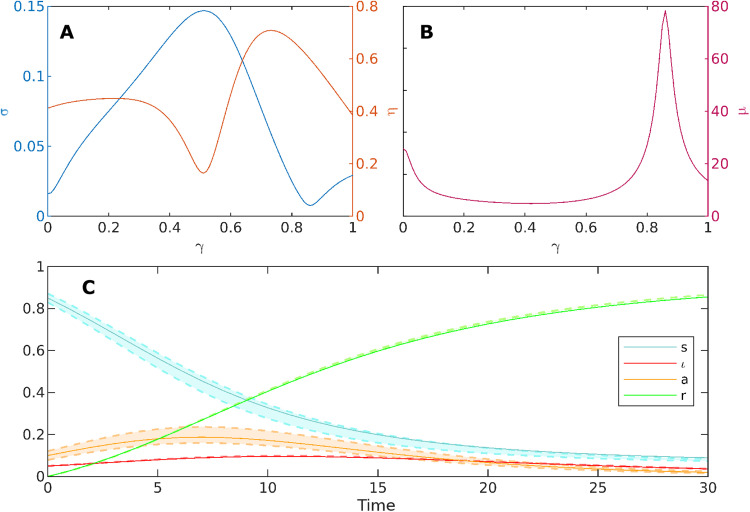
Practical identifiability of the SIAR model in Fig. 1 as a function of the fraction γ of asymptomatic new infectious individuals. (**A**) Sensitivity σ and η of measured and hidden variables, respectively, to the parameters of the model. (**B**) Sensitivity μ of the hidden variables to the measured ones. (**C**) State variables for unperturbed values of parameters (with γ = 0.86, solid line) and for a perturbation with ∥**δp** ∥ = 0.3 ∥ **p**∥ along the first eigenvector of M (dashed lines).

Figure 2A shows a nontrivial nonmonotonic dependence of our sensitivity measures, σ and η, on γ. The value of σ has a peak at γ = 0.51, in correspondence of which η takes its minimum value. This represents an optimal condition for practical identifiability, as the sensitivity to parameters of the measured variables is high, while that of the unmeasured ones is low, and this implies that the unknown quantities of the system (both the model parameters and the hidden variables) can be estimated with small uncertainty. On the contrary, for γ = 0.86, we observe a relatively small value of σ and a large value of η, meaning that the measured variables are poorly identifiable, and the unmeasured variables are sensitive to a variation of parameters. This is the worst situation in which the estimated parameters may substantially differ from the real values, and the hidden variables may experience large variations even for small changes in the parameters. Furthermore, the quantity μ, which measures the sensitivity of the hidden variables to the measured ones, reported in Fig. 2B, exhibits a large peak at the value of γ for which σ is minimal. This is due to the fact that the vector that determines μ is almost aligned with **v_1_**. When this holds, we have that μ = η/σ, which explains the presence of the spike in the μ curve. Similarly, the sensitivity μ takes its minimum almost in correspondence of the maximum of σ. The behavior of the model for γ = 0.86 is further illustrated in Fig. 2C, where the trajectories obtained in correspondence to the unperturbed values of the parameters, i.e., **m**(*t*, **p**) and **h**(*t*, **p**) (solid lines), are compared with the dynamics observed when **p** undergoes a perturbation with ∥**δp**∥ = 0.3∥**p**∥ along **v_1_** (dashed lines). The small sensitivity σ of the measured variables ι(*t*, **p**) and *r*(*t*, **p**) to parameters is reflected into perturbed trajectories that remain close to the unperturbed ones, whereas the large sensitivity η of the unmeasured variables *s*(*t*, **p**) and *a*(*t*, **p**) yields perturbed trajectories that significantly deviate from the unperturbed ones.

**Fig. 3.**
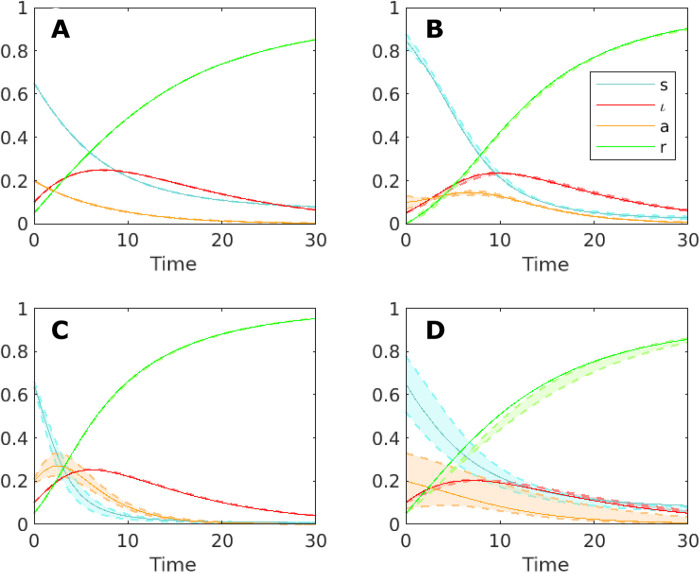
Four scenarios of identifiability for the SIAR model of Fig. 1. All panels show the system dynamics (solid line) and the evolution of the system when the vector of parameters undergoes a variation δ**p** such that ∥**δp** ∥ = 0.3 ∥ **p**∥ along the first eigenvector of M (dashed lines). (**A**) and (**C**) display configurations for which the observed variables (ι, *r*) are not sensitive to the variation, i.e., the model parameters are not identifiable, while (**B**) and (**D**) show the opposite case. Furthermore, (A) and (B) present scenarios for which the unobserved variables (*s, a*) are insensitive to the variation, meaning that they are predictable; vice-versa, (C) and (D) show the case in which the variables *s* and *a* are sensitive.

We now illustrate the different levels of identifiability that appear in the SIAR model for diverse settings of the parameters. Its analysis, in fact, fully depicts the more complete perspective on the problem of practical identifiability offered by simultaneously inspecting the sensitivity measures, σ and η. As the two sensitivity measures are not necessarily correlated, there can be cases for which a high identifiability of the measured variables to the parameters, i.e., large values of σ, corresponds to either a low or a high identifiability of the hidden variables to the parameters. Analogously, for other system configurations, in correspondence of small values of σ, namely, to nonidentifiable parameters, one may find large values of η, meaning that the hidden variables are nonidentifiable as well or, on the contrary, small values of η, indicating that the hidden variables are poorly sensitive to parameter perturbations. Together, four distinct scenarios of identifiability can occur, and all of them effectively appear in the SIAR model (Fig. 3): (A) low identifiability of the model parameters p and high identifiability of the hidden variables **h**, (B) high identifiability of both **p** and **h**, (C) low identifiability of both **p** and **h**, and (D) high identifiability of **p** and low identifiability of **h**. To illustrate them, we have considered four distinct configurations of the model (with parameters as given in Table 3 and illustrated in Materials and Methods) and, for each case study, compared the unperturbed trajectories to the perturbed ones, with the vector of parameters undergoing a variation ∥**δp** ∥ = 0.3 ∥ **p**∥ along **v_1_**. As regard cases (A) and (C), we have considered the vector of parameters to determine to be **p** = [ι(*t*), *a*(0), *r*(*t*), β_I_, β_A_, γ, α_IR_, α_AR_, α_AI_], while for the cases (B) and (D), we have **p** = [*a*(0), β_I_, β_A_, γ, α_AR_], which is the same choice of **p** adopted in Fig. 2. Figure 3 shows the results obtained for each parameter configuration. In each panel, the solid lines represent the unperturbed trajectories, while the dashed lines correspond to the perturbed dynamics. In cases (A) and (B), we see that, under the variation **δp**, the perturbed trajectories of the hidden variables remain close to the unperturbed dynamics. Hence, the hidden variables are highly identifiable. Conversely, in cases (C) and (D), the perturbed trajectories substantially differ from the unperturbed dynamics, meaning that the hidden variables are poorly identifiable as they are sensitive to a variation of the model parameters. As concerns the measured variables, in cases (A) and (C), the perturbed trajectories slightly differ from the unperturbed dynamics. Therefore, as the measured variables are insensitive to the perturbation δ**p**, the model parameters have a low degree of identifiability. On the other hand, in cases (B) and (D), the perturbation of the parameters significantly affects the trajectories of the measured variables, meaning that the set of parameters reproducing the observed data is more identifiable.

Last, Table 1 illustrates the values of the sensitivity measures σ, η, and μ for each case. In particular, case (C) represents the worst scenario as the value of σ is relatively small, meaning that the model parameters **p** are poorly identifiable, and the value of η is large, indicating a high sensitivity of the hidden variables to the parameters. Conversely, the best scenario is represented by case (B), for which both the model parameters and the hidden variables are highly identifiable as the value of σ is large compared to the other cases, while the value of η remains relatively small.

**Table 1.** Values of σ, η, and μ for the four configurations of the SIAR model shown in Fig. 3.

	**Case A**	**Case B**	**Case C**	**Case D**
σ	0.0096	0.15	0.013	0.091
η	0.012	0.16	0.36	1.4
μ	34	5.2	29	15

### Poorly identifiable models may provide unreliable predictions when the parameters are estimated from data

So far, we have illustrated how variations on the parameters affect the trajectories of measurable and hidden variables under different degrees of identifiability. However, a high sensitivity of the hidden variables to measured ones has relevant practical consequences, especially when the parameters are unknown and need to be fitted from data. In these conditions, a small uncertainty in the measurable variables due to the presence of noise in the data can propagate markedly and make the prediction of the hidden variables unreliable. Hence, in this section, we study how the lack of practical identifiability can affect the predictions of the SIAR model when this is fitted to empirical data. The reliability of the model predictions has been investigated by means of two different fitting techniques: a least square error minimization procedure (*42*), which provides a point estimate of the parameters, and a Bayesian inference approach (*43*), which, conversely, gives an estimate of the probability distribution in the parameter space. To carry out the numerical analysis, we consider the model under the same settings (i.e., fixing the values of the six parameters and the initial values of three variables) as those adopted in case (C) in the previous section, which correspond to the case of low identifiability of both the parameters and the hidden variables. We then generate from such a model a synthetic dataset of trajectories, which we fit using the two approaches mentioned above (see Materials and Methods for further details). All the model parameters are considered unknown and thus need to be determined through the fit.

**Fig. 4.**
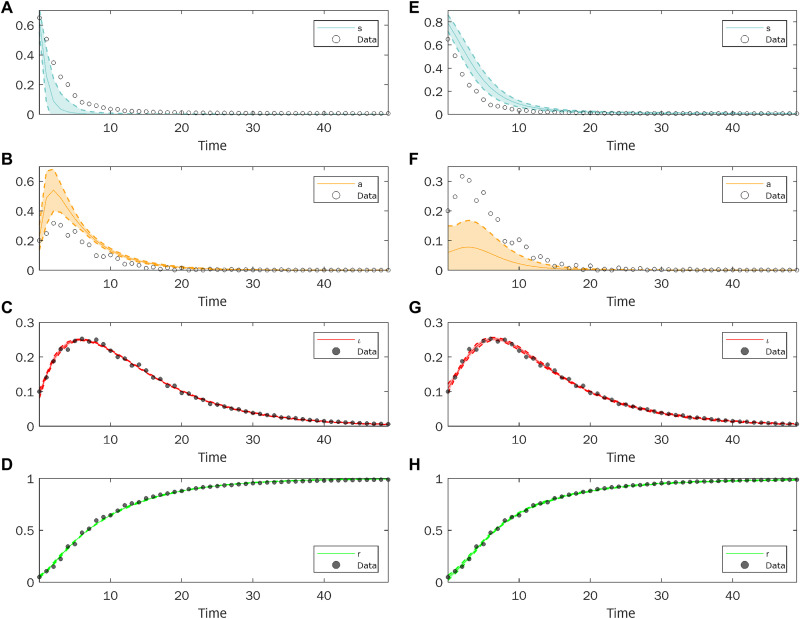
Dynamics of the SIAR model when two different methods to estimate the model parameters from data are used. (**A** to **D**) display the results obtained by a least square error minimization procedure, while (**E** to **H**) show the outcome of Bayesian inference. The time evolution of both measured and hidden variables (solid lines) is reported together with the data. Data are shown with different markers if they pertain to measured variables (full circles, used for fitting) or unmeasured ones (empty circles, not used for fitting).

First, we consider the least square error minimization approach. To show how, because of the lack of identifiability of the model, significant variations in the dynamics of the hidden variables can be obtained when fitting the measured variables, we have performed the following analysis. As the estimation procedure (based on a nonlinear optimization algorithm; see Materials and Methods) starts from an initial guess of the fitting parameters, indicated as **p_0_**, instead of fitting a single set of values, we have repeated the procedure under the very same conditions of the algorithm, for 500 runs, randomly selecting **p_0_** from a Gaussian distribution centered on a fixed point of the parameter space and with variance equal to 0.25. We then discarded those runs yielding a fitting error *d* > 0.015, which corresponds to a relative error of 2.5%, thus keeping a total of 65 sets of parameters fitting the measured variables with a similar value of the error. The fact that different sets of parameters are obtained in this way may indicate that the error function has several local minima. Figure 4 (A to D) displays the average trajectories (over the 65 sets of parameters) of the four state variables (solid lines) and the respective regions where 95% of the trajectories lie (shadowed area). While the dynamics of the measured variables (Fig. 4, C and D) produced by the SIAR model are in very good agreement with the data, the same is not true for the temporal evolution of the hidden variables (Fig. 4, A and B). In particular, the number of newly asymptomatic infected individuals is substantially overestimated.

We now consider the Bayesian inference approach. To implement it, we used the Delayed Rejection Adaptive Metropolis (DRAM) (*44*), a Markov Chain Monte Carlo (MCMC) algorithm, with settings as described in Materials and Methods. In particular, since we have here assumed to have no a priori information on the value of the model parameters, we have considered uniform prior probability distributions, representing the less informative conditions for the model (further details are given in Materials and Methods). Figure 4 (E to H) shows the temporal evolution of the SIAR variables. Solid lines represent the average trajectory obtained by sampling 500 sets of parameters from the posterior distributions, while the shadowed areas indicate the regions where 95% of the trajectories lie.

Similarly, to the case of the least square minimization, while the dynamics of the measured variables (Fig. 4, G and H) is in a good agreement with the synthetic data, the prediction of the hidden variables (Fig. 4, E and F) is not. At variance with the previous example, the number of newly asymptomatic infected individuals is here largely underestimated. Hence, these results indicate that the lack of identifiability can lead to unreliable results even when a Bayesian approach is adopted.

In the analysis presented in this section, we have assumed to have no a priori information on the values of the model parameters. Therefore, when performing the least square error minimization, we have extracted all the parameters from fitting, while, following the same reasoning, we have chosen a uniform prior probability distribution in the Bayesian approach. When instead we have strong a priori knowledge of a disease, this can be used to inform the models, by fixing the values of certain parameters while estimating the others, in the case of the least square error method, or by considering more informative prior distributions, in the case of Bayesian inference. When a priori information on the values of the parameters can be obtained, for instance, through medical and biological studies, the model predictions are expected to become less affected by uncertainty. The analysis of the sensitivity matrices (see also Materials and Methods) confirms this expectation as we have demonstrated that additional knowledge of the parameters, or the measurement of a hidden variable, can reduce the sensitivity to the measured variables, thus improving the reliability of the prediction. However, there are cases in which a priori knowledge is not available, and the analysis of identifiability becomes crucial. As an example, one could estimate the percentage of asymptomatic individuals according to serological surveys or to longitudinal studies. However, these data can be unavailable at an early stage of an epidemic outbreak (*45*, *46*), preventing their use to inform the epidemiological models. These considerations hallmark once again the need for a synergistic approach to study newly discovered infectious diseases and stress the importance of assessing the reliability of mathematical modeling when the amount of available information is limited.

### Lack of identifiability in COVID-19 modeling prevents reliable predictions

**Fig. 5.**
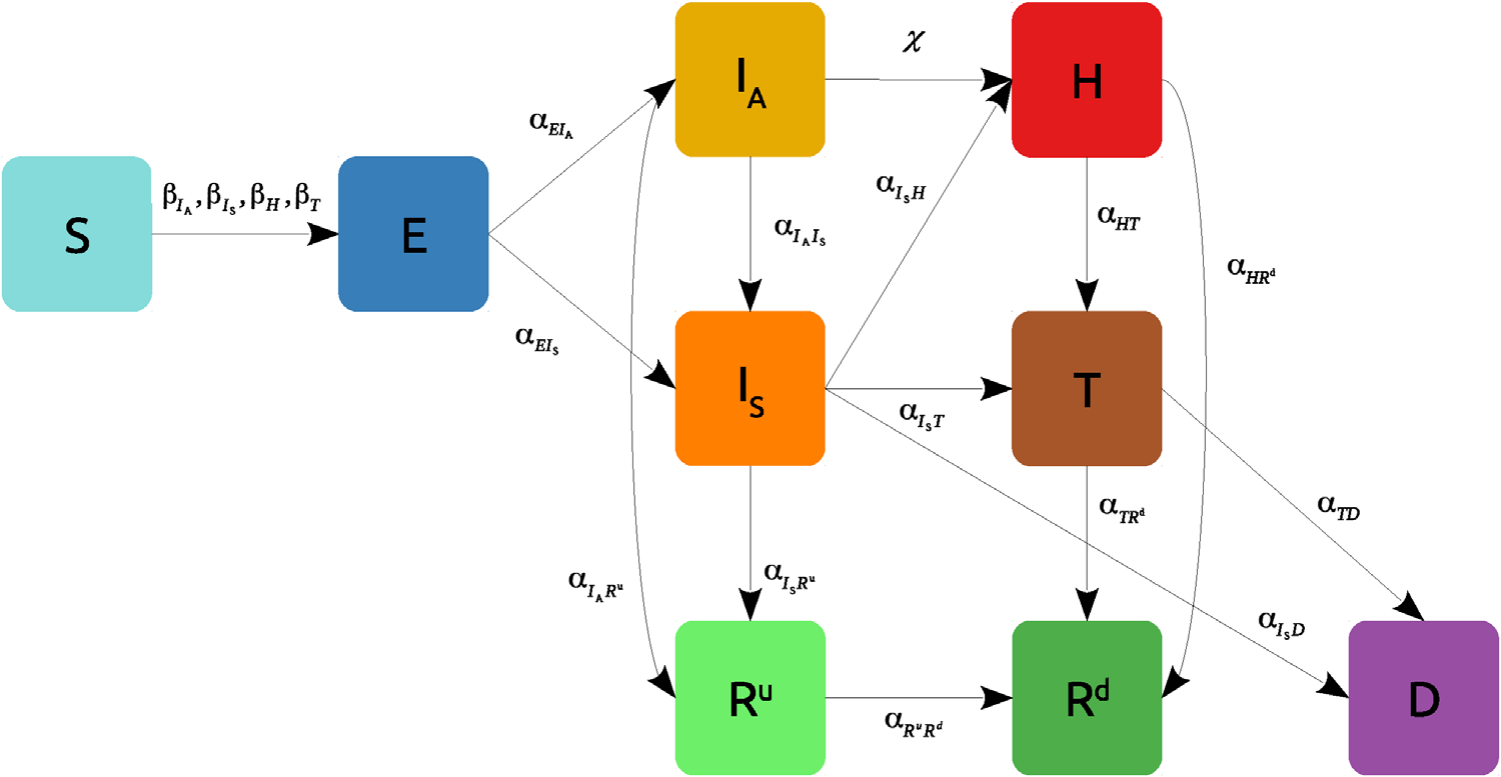
Graphical representation of a nine-compartment model for the propagation of COVID-19 (see also Eq. 24 in Materials and Methods).

As a second application, we show the relevance of the problem of practical identifiability in the context of COVID-19 pandemic modeling. We consider a realistic model (Fig. 5) of the disease propagation, that is a variant of the SIDARTHE model (*16*) and is characterized by nine compartments accounting respectively for susceptible (*S*), exposed (*E*), undetected asymptomatic (*I*_A_), undetected symptomatic (*I*_S_), home isolated (*H*), treated in hospital (*T*), undetected recovered (*R*^u^), detected recovered (*R*^d^), and deceased (*D*) individuals. Following the study of Giordano *et al*. (*16*), to account for the different nonpharmaceutical interventions and testing strategies issued during the COVID-19 outbreak in Italy (*47*, *48*), the model parameters have been considered piece-wise constant and estimated using nonlinear optimization by fitting of the official data provided by the Civil Protection Department (*49*). As the dataset provides the evolution in time of the daily number of home isolated, hospitalized, detected recovered, and deceased individuals, we have considered four measured and five hidden variables in the model, namely, **m** ≡ [*H*, *T*, *R*^d^, *D*] and **h** ≡ [*S*, *E*, *I*_A_, *I*_S_, *R*^u^].

It is here worth discussing an important issue that concerns the model parameters. We have considered that different policy strategies affect, according to their nature, only specific parameters. In particular, we have assumed that a change in the containment strategy leads to a variation in the transmission rates β, while an adjustment in the testing strategy affects the values of the parameters α_*I*_S_*H*_, α*_HT_*, and α*_HR^d^_* (further details are reported in Materials and Methods). As both the containment and the testing strategies in Italy have frequently changed during the pandemic, most of the parameters to estimate consist of transmission and detection rates. While other parameters, such as the death or the recovery rates, can be derived from the current literature (*50*), very limited information is available on the transmission and detection rates that are difficult to measure directly and, therefore, need to be estimated by fitting available data. Hence, as in (*16*), we have assumed all the model parameters to be unknown.

**Fig. 6.**
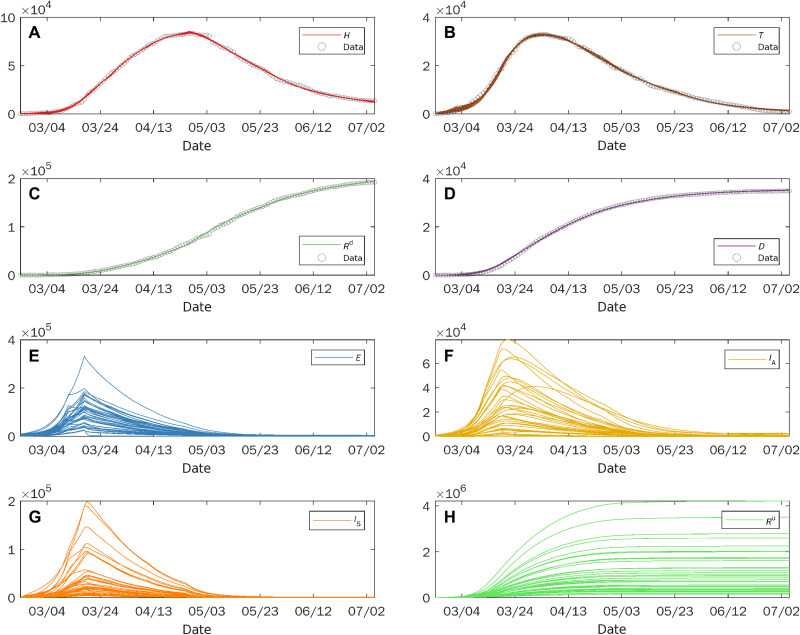
Modeling the COVID-19 outbreak in Italy. The evolution of both measured (**A** to **D**) and hidden variables (**E** to **H**) of the model in Fig. 5 (solid lines) is reported together with the official data from the Civil Protection Department (circles).

To show how significant variations in the evolution of the hidden variables can arise when fitting the measured variables, we have performed a numerical analysis similar to the one of the previous section. Again, rather than fitting a single set of values, we have repeated the minimization procedure under the same conditions of the algorithm, for 500 runs, randomly selecting the initial guess **p_0_** from a Gaussian distribution centered on a fixed point of the parameter space, with variance equal to 0.25. We discarded the runs yielding a fitting error *e* > 900, corresponding to a relative error of 1.4%, thus keeping a total of 40 sets of parameters. Figure 6 shows the dynamical trajectories that we have obtained for each of the 40 sets of parameters (solid lines). Both measured (Fig. 6, A to D) and hidden (Fig. 6, E to H) variables are reported. While the time evolution of the measured variables produced by the model is in very good agreement with the empirical data, reported as circles in Fig. 6, significant differences in the trend of the hidden variables appear. A large variability is observed, confirming that the lack of identifiability yields a high sensitivity of the hidden variables to the measured one.

These findings have relevant implications. The large uncertainty on the size of the asymptomatic population makes questionable the use of the model as a tool to decide the policies to adopt.

## DISCUSSION

The practical identifiability of a dynamical model is a critical, but often neglected, issue in determining the reliability of its predictions. In this paper, we have introduced a novel framework to quantify: (i) the sensitivity of the dynamical variables of a given model to its parameters, even in the presence of variables that are difficult to access empirically and (ii) how changes in the measured variables affect the evolution of the unmeasured ones.

The measures we have proposed are easy to compute and enable to assess, for instance, if and when the model predictions on the unmeasured variables are reliable or not, even in the cases in which the parameters of the model can be fitted with high accuracy from the available data.

As we have shown with a series of case studies, practical identifiability can critically affect the predictions of even very refined epidemic models introduced for the description of COVID-19, where dynamical variables, such as the population of asymptomatic individuals, are impossible or difficult to measure. This by no means should question the importance of these models—in that they enable a scenario analysis, otherwise impossible to carry out, and a deeper understanding of the spreading mechanisms of a novel disease—but should hallmark the relevance of a critical analysis of the results that takes into account sensitivity measures. It also highlights the importance of cross-disciplinary efforts that can provide a priori information on some of the parameters, ultimately improving the reliability of a model (*8*, *24*).

A problem related to the one studied in our paper is that of observability, which investigates how to reconstruct the internal state of a system from measurements on the input and output, under the hypothesis that the model and its parameters are known (*51*, *52*). Techniques based on the observability problem are clearly extremely important and may be applied, for instance, to derive the time evolution of asymptomatic individuals from measurements on infected and recovered individuals, when it is possible to develop a fully observable model with known parameters.

## MATERIALS AND METHODS

### The sensitivity matrices and their properties

The sensitivity matrices considered in this paper are given by

$$M_{\mathrm{ij}}=\int_{0}^{\infty }\frac{\partial m^{T}}{\partial p_{i}}\frac{\partial m}{\partial p_{j}}\text{dt};\;H_{\mathrm{ij}}=\int_{0}^{\infty }\frac{\partial h^{T}}{\partial p_{i}}\frac{\partial h}{\partial p_{j}}\text{dt}$$ (6)

where the vector functions **m** = **m**(*t*, **p**) and **h** = **h**(*t*, **p**) are obtained integrating system in eq. 1. The derivative of measurable and hidden variables with respect to the parameters **p**

$$m_{i}\equiv \partial m/\partial p_{i},\;h_{i}\equiv \partial h/\partial p_{i}$$

can be obtained by integrating the system

$$\begin{array}{cc} \frac{dm_{i}}{\text{dt}} & =\frac{\partial f}{\partial m}\cdot m_{i}+\frac{\partial f}{\partial h}\cdot h_{i}+\frac{\partial f}{\partial p_{i}}\\ \frac{dh_{i}}{\text{dt}} & =\frac{\partial g}{\partial m}\cdot m_{i}+\frac{\partial g}{\partial h}\cdot h_{i}+\frac{\partial g}{\partial p_{i}} \end{array}$$ (7)

where *i* = 1, …*n_p_*.

The numerical evaluation of the sensitivity matrices is carried out first by integrating system in eq. 7 (for this step we use a fourth-order Runge-Kutta solver with adaptive step size control), resampling the trajectories with a sampling period of 1 day, and then performing a discrete summation over the sampled trajectories. Moreover, integration is carried out over a finite time interval [0, τ], with large enough τ. In the context of our work, as we have considered SIR (susceptible infected removed)-like epidemic models, we set the value of τ such that the system has reached a stationary state, i.e., the epidemic outbreak has ended, as every infected individual has eventually recovered (or dead, depending on the model).

We now present an important property of the sensitivity matrices. We will only take into account the set of measured variables **m**, as similar considerations can be made for the hidden variables. Let us assume to be able to measure only a single variable, so that the vector **m** collapses into a scalar function, which we call *m*_1_(*t*). In this case, the element M*_ij_* of the sensitivity matrix would be simply given by

$${\left(M\right)}_{\mathrm{ij}}=\int_{0}^{\infty }\frac{\partial m_{1}}{\partial p_{i}}\frac{\partial m_{1}}{\partial p_{j}}\text{dt}$$ (8)

Let us call this sensitivity matrix M_1_.

Consider now a larger set of measured variables **m** = (*m*_1_, *m*_2_, …, *m_n_m__*). The quantity ∂**m***^T^*/∂*p_i_*∂**m**/*∂p_j_* in Eq. 6 is given by

$$\frac{\partial m^{T}}{\partial p_{i}}\frac{\partial m}{\partial p_{j}}=\frac{\partial m_{1}}{\partial p_{i}}\frac{\partial m_{1}}{\partial p_{j}}+\frac{\partial m_{2}}{\partial p_{i}}\frac{\partial m_{2}}{\partial p_{j}}+\ldots +\frac{\partial m_{n_{m}}}{\partial p_{i}}\frac{\partial m_{n_{m}}}{\partial p_{j}}$$ (9)

Therefore, integrating over time in the interval [0, ∞ ] and given the linearity property of the integrals, we find that the sensitivity matrix M of the set of the measured variables is given by the sum of the sensitivity matrices of the single measured variables. Formally, we have that

$$M=M_{1}+M_{2}+\ldots +M_{n_{m}}$$ (10)

This property of the sensitivity matrices is useful to demonstrating how measuring a further variable affects the sensitivity measures σ and μ, as discussed in the following subsection and in Results. Last, because matrices M and H are positive semidefinite, their eigenvalues are nonnegative. For any positive semidefinite matrix A of order *m*, we shall denote its eigenvalues as 0 ≤ λ_1_(A) ≤ λ_2_(A) ≤ … ≤ λ*_m_*(A).

### Sensitivity measures and their properties

Here, we discuss in more detail the sensitivity measures introduced in Results. First, we want to propose a measure to quantify the practical identifiability of the model parameters given the measured variables. To do this, we need to evaluate the sensitivity of the trajectories of the measured variables to a variation of the model parameters. If this sensitivity is small, then different sets of parameters will produce very similar trajectories of the measured variables, meaning that the parameters themselves are poorly identifiable. In particular, as a measure of the parameters identifiability, we can consider the worst scenario, namely, the case in which the perturbation of the parameters minimizes the change in the measured variables. This happens when the variation of the model parameters δ**p** is aligned along the eigenvector **v**_1_ of M corresponding to the minimum eigenvalue λ_1_(M). Given the definition of M, we have that $∥\delta m∥=\sqrt{\lambda_{1}(M)}∥\delta p∥$; hence, we can consider the quantity

$$\sigma =\sqrt{\lambda_{1}(M)}$$ (11)

as an estimate of the sensitivity of the measured variables to the parameters. Note that, here and in the rest of the paper, ∥**v**∥ denotes the Euclidean norm of a finite dimensional vector **v**, ∥**v**∥^2^ = **v** · **v**, while for a function **u**(*t*), ∥**u**∥ denotes the *L*^2^ norm of **u** in [0, ∞ ], i.e.,$∥u{∥}^{2}=\int_{0}^{\infty }u\cdot u\;\text{dt}$.

Let us now focus on the hidden variables **h**. In general, as the hidden variables are not directly associated to empirical data, the largest uncertainty on the hidden variables is obtained in correspondence of a variation of the parameters along the eigenvector of H associated to the largest eigenvalue, namely, $\lambda_{n_{p}}$(H).

Hence, to quantify the sensitivity of the hidden variables to the parameters, one may consider

$$\eta_{\mathrm{MAX}}=\sqrt{\lambda_{n_{p}}(H)}$$ (12)

However, it is crucial to note that the hidden variables ultimately depend on the parameters of the model, which are estimated by fitting data that are available for the measured variables only. As a consequence, it is reasonable to consider a quantity that evaluates how the uncertainty on the model parameters (determined by the uncertainty of the measured variables and by their sensitivity to the parameters) affects the identifiability of the hidden variables. Therefore, as a measure of the sensitivity of the hidden variables to the parameters, we consider

$$\eta^{2}=\frac{\delta p_{1}^{T}H\delta p_{1}}{\delta p_{1}^{T}\delta p_{1}}$$ (13)

where δ**p**_1_ is a perturbation of the parameters along the eigenvector **v**_1_ of M corresponding to the minimum eigenvalue λ_1_(M). Note that, when **v**_1_ and the eigenvector of H corresponding to the largest eigenvalue $\lambda_{n_{p}}$(H) are aligned, by definition, we have η = η*_MAX_*.

Last, we want to define a quantity to estimate how much the hidden variables are perturbed given a variation of the measured ones. In particular, as a measure of the sensitivity of the hidden variables to the measured variables, we consider the maximum perturbation of the hidden variables given the minimum variation of the measured ones, which is

$$\mu^{2}=\underset{∥\delta p∥=1}{\text{max}}\frac{\delta p^{T}H\delta p}{\delta p^{T}M\delta p}$$ (14)

Note that μ^2^ can be computed considering the following generalized eigenvalue problem

$$Hu_{k}=\lambda_{k}Mu_{k}$$ (15)

where H and M are the sensitivity matrices for the hidden and the observed variables respectively, and λ*_k_* = λ*_k_*(M, H) denotes the *k*-th generalized eigenvalue of matrices M and H. We will denote by $\lambda_{n_{p}}$ the largest generalized eigenvalue and **u** the corresponding generalized eigenvector. Note that, since both matrices are symmetric, if **u** is a right eigenvector, then **u***^T^* is a left eigenvector. Multiplying each member of the equation by **u***^T^* and dividing by **u***^T^*M**u**, we obtain

$$\lambda_{n_{p}}=\frac{u^{T}Hu}{u^{T}Mu}=\underset{∥v∥=1}{\text{max}}\frac{v^{T}Hv}{v^{T}Mv}$$ (16)

where one can recognize the definition of μ^2^ provided in Eq. 14. In other words, μ^2^ represents the largest eigenvalue of the matrix M^−1^H.

It is worth noting two aspects about the sensitivity measure μ. First, given the definitions in Eqs. 11 and 12, for any δ**p** with ∥δ**p**∥ = 1, we have that $\delta p^{T}H\delta p\leq \eta_{\mathrm{MAX}}^{2}$ and δ**p***^T^*Mδ**p** ≥ σ^2^. As a consequence, we have that

$$\mu^{2}\leq \frac{\eta_{\text{MAX}}^{2}}{\sigma^{2}}$$ (17)

Second, when the vector δ**p** that determines μ is aligned with the eigenvector **v_1_** of M, it is possible to express μ in terms of the sensitivity measures σ and η. When δ**p** = ∥ δ**p** ∥ **v**_1_ = v_1_, recalling definitions in Eqs. 11 and 13, one obtains $v_{1}^{T}Mv_{1}=\sigma^{2}$, while $v_{1}^{T}Hv_{1}=\eta^{2}$, from which it follows

$$\mu =\frac{\eta }{\sigma }$$ (18)

In addition, we note that if **v**_1_ and the eigenvector of H corresponding to its largest eigenvalue are aligned, one obtains that μ = η*_MAX_*/σ, which is the maximum value for the sensitivity measure μ.

We now demonstrate that the sensitivity of the hidden variables to the measured ones, μ^2^, decreases as we measure one further variable. Let us assume now that we are able to measure one further variable, thus increasing the size of the set of measured variables to *n_m_*′ = *n_m_* + 1 and, correspondingly, reducing that of the unmeasured variables to *n_h_*^′^ = *n_h_* − 1. Given the property in Eq. 10, the new sensitivity matrices can be written as M^′^ = M + M_1_ and H^′^ = H − M_1_, where by M_1_, we denote the sensitivity matrix for the newly measured variable. The new generalized eigenvalue problem is

$$H\prime u\prime =\lambda \prime M\prime u\prime \Leftrightarrow (H-M_{1})u\prime =\lambda \prime (M+M_{1})u\prime$$ (19)

where, for simplicity, we have denoted by λ^′^ the largest generalized eigenvalue of matrices M^′^ and H^′^.

Left multiplying by **u**^′*T*^ and dividing by **u**^′*T*^M**u**^′^, we obtain

$$\begin{array}{c} \lambda_{n_{p}}=\frac{u^{T}Hu}{u^{T}Mu}\geq \frac{{u\prime }^{T}Hu\prime }{{u\prime }^{T}Mu\prime }=\frac{{u\prime }^{T}H’u\prime +{u\prime }^{T}M_{1}u\prime }{{u\prime }^{T}Mu\prime -{u\prime }^{T}M_{1}u\prime }\geq \\ \frac{{u\prime }^{T}H’u\prime }{{u\prime }^{T}M^{’}u\prime }=\lambda \prime \end{array}$$ (20)

where the first inequality comes from the definition of $\lambda_{n_{p}}$, while the second comes from the fact that H, M, H^′^, M^′^, and M_1_ are positive semidefinite. In short, we find that $\lambda_{n_{p}}$ ≥ λ′, meaning that, by measuring one variable, the sensitivity of the hidden variables to the measured ones decreases.

### SIAR model and setup for numerical analysis

The SIAR model of Fig. 1 is described by the following equations

$$\left\{ \begin{array}{l} \overset{\cdot }{s}=-s(\beta_{I}\iota +\beta_{A}a)\\ \overset{\cdot }{\iota }=(1-\gamma )s(\beta_{I}\iota +\beta_{A}a)+\alpha_{\mathrm{AI}}a-\alpha_{\mathrm{IR}}\iota \\ \overset{\cdot }{a}=\gamma s(\beta_{I}\iota +\beta_{A}a)-(\alpha_{\mathrm{AI}}+\alpha_{\mathrm{AR}})a\\ \overset{\cdot }{r}=\alpha_{\mathrm{IR}}\iota +\alpha_{\mathrm{AR}}a \end{array}\right.$$ (21)

where *s*(*t*), ι(*t*), *a*(*t*), and *r*(*t*) represent population densities, i.e., *s*(*t*) = *S*(*t*)/*N*, ι(*t*) = *I*(*t*)/*N*, *a*(*t*) = *A*(*t*)/*N*, and *r*(*t*) = *R*(*t*)/*N*, where *S*(*t*), *I*(*t*), *A*(*t*), and *R*(*t*) represent the number of susceptible, infectious, asymptomatic, and recovered individuals, and *N* is the size of the population, so that *s*(*t*) + ι(*t*) + *a*(*t*) + *r*(*t*) = 1. Here, β*_I_* and β*_A_* are the transmission rates for the symptomatic and the asymptomatic individuals, respectively, γ is the probability for newly infected individuals to show no symptoms, α*_AI_* is the rate at which asymptomatic individuals become symptomatic, and α*_IR_* and α*_AR_* are the recovery rates for the two infectious populations. Note that all these parameters are positive quantities.

Asymptomatic individuals are difficult to trace as the individuals themselves could be unaware about their state. As a consequence, we assume that the density of asymptomatic individuals is not measurable, while the densities of symptomatic and recovered individuals are measured variables. According to the notation introduced in Eq. 1, we therefore have that **m** ≡ [ι, *r*] and **h** ≡ [*s*, *a*]. Note that, as a first approximation, here, we assume to be able to trace the asymptomatic individuals once they recover.

The results presented in Fig. 2 have been obtained considering the following setup. As the number of symptomatic infectious and recovered individuals are considered measurable, we have assumed that the initial conditions ι(0), r(0), and the rate of recovery α*_IR_* are known parameters. Second, we have supposed to be able to measure, for instance, through backward contact tracing the rate at which asymptomatic individuals develop symptoms, i.e., α*_AI_*. Hence, the vector of parameters to determine by calibrating the model is given by **p** = [*a*(0), β*_I_*, β*_A_*, γ, α*_AR_*]. Table 2 displays the value of the model parameters used to obtain the results shown in Fig. 2.

**Table 2.** Values of the model parameters used for the case study in Fig. 2.

**ι_0_**	***a*0**	***r*0**	**β*_I_***	**β*_A_***	**α*_IR_***	**α*_AR_***	**α*_AI_***
0.05	0.1	0	0.6	0.3	0.1	0.2	0.03

For the analysis of the four scenarios considered in Fig. 3, the values of the model parameters have been set as given in Table 3. Furthermore, to better contrast the results arising in the different case studies, in (A) and (C), we have considered **p** = [ι(0), *a*(0), *r*(0), β*_I_*, β*_A_*, γ, α*_IR_*, α*_AR_*, α*_AI_*], while in (B) and (D), we have set **p** = [*a*(0), β*_I_*, β*_A_*, γ, α*_AR_*].

**Table 3.** Values of the model parameters used for the case study in Fig. 3.

	**ι_0_**	***a*0**	***r*>0**	**β*_I_***	**β*_A_***	**γ**	**α*_IR_***	**α*_AR_***	**α*_AI_***
Case A	0.1	0.2	0.05	0.3	0.4	0.26	0.1	0.2	0.03
Case B	0.05	0.1	0	0.6	0.3	0.51	0.1	0.2	0.03
Case C	0.1	0.2	0.05	0.6	0.8	0.77	0.1	0.2	0.1
Case D	0.1	0.2	0.05	0.3	0.4	0.53	0.1	0.2	0.03

### Estimating the parameters of the SIAR model

The SIAR model parameters have been estimated from data by adopting two different approaches, i.e., a nonlinear least square error minimization and a Bayesian inference. To generate a synthetic dataset, we have integrated the deterministic model in Eq. 21. To mimic measurement errors, we have adopted the following procedure. First, we compute $\bar{r}(t)$ by adding to *r*(*t*) a uniform noise in the interval (− δ*_t_*/2, δ*_t_*/2), where δ*_t_* = ∣*r*(*t* + 1) − *r*(*t*)∣, checking that the synthetic time series remains monotonically nondecreasing. We have then generated the data $\bar{s}(t)$ in a similar fashion, this time controlling that the synthetic time series remains monotonically nonincreasing. To generate the data $\bar{\iota }(t)$, we have added a Gaussian noise with zero mean and standard deviation (SD) equals to 3% to the time series, making sure that $\bar{s}(t)+\bar{\iota }(t)+\bar{r}(t)\leq 1$. Last, the data $\bar{a}(t)$ have been evaluated using the fact that $\bar{s}(t)+\bar{\iota }(t)+\bar{a}(t)+\bar{r}(t)=1$.

The integration of Eq. 21 has been carried out by using the lsoda ordinary differential equation (ODE) solver (*53*, *54*) and then resampling the data with a sampling period of one time unit. We assumed that the density of asymptomatic individuals *a* is not measurable, while the densities of symptomatic and recovered individuals, i.e., ι and *r*, are measured variables.

As regard to the least square error minimization approach, the model parameters have been estimated using a nonlinear optimization procedure (implemented via the function fmincon in MATLAB) with the following objective function to minimize

$$d=\sqrt{\frac{1}{2\tau }\sum_{k=1}^{\tau }({\left(\iota (k)-\bar{\iota }(k)\right)}^{2}+{\left(r(k)-\bar{r}(k)\right)}^{2})}$$ (22)

where $\bar{\iota }(k)$ and $\bar{r}(k)$ with *k* = 1, …, τ (with τ = 50) represent the noisy synthetic time series of the densities of infectious and recovered individuals, respectively, while ι(*k*) and *r*(*k*) are the values of the corresponding variables obtained from the integration of Eq. 21.

The core idea of Bayesian inference is to provide an a posteriori probability distribution for the model parameter vector, **p**, given an a priori probability distribution on the value of **p** and a likelihood function, which quantifies the goodness of a model in reproducing empirical data D. The relationship between these is given by the Bayes’ theorem, which reads

$$\pi (p|D)=\frac{L(D|p)\pi (p)}{\int_{p}L(D|p)\pi (p)dp}$$ (23)

where π(**p**) indicates the prior distribution, ℒ(𝒟∣**p**) the likelihood, and π(**p**∣𝒟) the posterior distribution. Usually, it is not possible to evaluate analytically the integral appearing in the denominator, especially when a large number of parameters are considered. Therefore, one relies on MCMC algorithms, which allow one to approximate of the posterior distribution. The MCMC algorithm we used to implement the Bayesian inference is the DRAM (*44*). As the likelihood function ℒ(𝒟∣**p**), we have considered the root mean square error, evaluated on the measurable variables only, i.e. ι(*t*) and *r*(*t*), which corresponds to Eq. 22, namely, to the objective function of the nonlinear optimization procedure. As we have assumed to have no a priori knowledge of the values of the model parameters, for the Bayesian inference, we have considered uniform prior probability distributions, which are the simplest and least informative choice (*55*, *56*). Flat priors do not require any additional information apart from setting the interval of possible parameter values. These intervals have been defined taking into account the following considerations. On the one hand, we have the initial conditions of the dynamical variables. As in the SIAR model, these represent population densities, we can assume the uniform prior distribution for their initial conditions to be defined in the interval [0,1]. Similarly, the parameter γ, which indicates the fraction of newly infected individuals not developing symptoms, can be assumed to be defined in the same interval. As regard the remaining parameters, since we have assumed to not have any other information except for the fact that they are positive quantities, we can consider the uniform distribution to be extended in the interval [0, ∞ ].

### Nine-compartment model for COVID-19

The nine-compartment model of Fig. 5 can be considered as a variant of the SIDARTHE model (*16*). It is characterized by the presence of an incubation state, in which the individuals have been exposed to the virus (*E*) but are not yet infectious, and by infectious individuals, that, in addition to being symptomatic or asymptomatic, can be either detected or undetected. The model, therefore, includes four classes of infectious individuals: undetected asymptomatic (*I*_A_), undetected symptomatic and pauci-symptomatic (*I*_S_), home isolated (*H*, corresponding to detected asymptomatic and pauci-symptomatic), and treated in hospital (*T*, corresponding to detected symptomatic). Last, removed individuals can be undetected (*R*^u^), detected (*R*^d^), or deceased (*D*).

The model dynamics is described by the following equations

$$\left\{ \begin{array}{l} \overset{\cdot }{S}=-S(\beta_{I_{A}}I_{A}+\beta_{I_{S}}I_{S}+\beta_{H}H+\beta_{T}T)/N\\ \overset{\cdot }{E}=S(\beta_{I_{A}}I_{A}+\beta_{I_{S}}I_{S}+\beta_{H}H+\beta_{T}T)/N\\ -(\alpha_{{\text{EI}}_{A}}+\alpha_{{\text{EI}}_{S}})E\\ {\overset{\cdot }{I}}_{A}=\alpha_{{\text{EI}}_{A}}E-(\alpha_{I_{A}I_{S}}+\alpha_{I_{A}R^{u}})I_{A}-\chi I_{A}\\ \dot{I_{S}}=\alpha_{{\text{EI}}_{S}}E+\alpha_{I_{A}I_{S}}I_{A}\\ -(\alpha_{I_{S}H}+\alpha_{I_{S}T}+\alpha_{I_{S}R^{u}}+\alpha_{I_{S}D})I_{S}\\ \overset{\cdot }{H}=\alpha_{I_{S}H}I_{S}+\chi I_{A}-(\alpha_{\text{HT}}+\alpha_{{\text{HR}}^{d}})H\\ \overset{\cdot }{T}=\alpha_{I_{S}T}I_{S}+\alpha_{\text{HT}}H-(\alpha_{TR^{d}}+\alpha_{\mathrm{TD}})T\\ {\overset{\cdot }{R}}^{u}=\alpha_{I_{A}R^{u}}I_{A}+\alpha_{I_{S}R^{u}}I_{S}\\ {\overset{\cdot }{R}}^{d}=\alpha_{{\text{HR}}^{d}}H+\alpha_{{\mathrm{TR}}^{d}}T\\ \overset{\cdot }{D}=\alpha_{I_{S}D}I_{S}+\alpha_{\mathrm{TD}}T \end{array}\right.$$ (24)

where the state variables represent the number of individuals in each compartment, *N* = 60 · 10^6^ and *S* + *E* + *I_A_* + *I_S_* + *H* + *T* + *R^u^* + *R^d^* + *D* = *N*. The official data on the spreading of COVID-19 in Italy made available by the Civil Protection Department [Dipartimento della Protezione Civile, (*49*)] provide information only on four of the nine compartments of the model, namely, the home isolated (*H*), hospitalized (*T*), detected recovered (*R*^d^), and deceased individuals (*D*). These compartments constitute the set of the measured variables, while the other variables have to be considered as hidden, i.e., **m** ≡ [*H*, *T*, *R*^d^, *D*] and **h** ≡ [*S*, *E*, *I*_A_, *I*_S_, *R*^u^].

All the parameters appearing in (*24*) are considered unknown; thus, they need to be determined through fitting the model to the available data. It should also be noted that, as many nonpharmaceutical interventions have been issued/lifted, and the testing strategy has been changed several times over the course of the epidemics (*47*, *48*), not all parameters can be considered constant in the whole period used for the fitting. Hence, similarly to (*16*), we have divided the whole period of investigation (which in our case ranges from 24 February to 06 July 2020) into different windows, within each of which the parameters are assumed to be constant. In each time window, one allows only some parameters to vary according to what is reasonable to assume will be influenced by the government intervention during that time window.

We distinguish two kinds of events that may require an adaptation of the model parameters. On the one hand, there are the nonpharmaceutical containment policies aimed at reducing the disease transmission. When these interventions are issued, the value of the parameters β may vary. On the other hand, the testing strategy, which affects the probability of detecting infected individuals, was also not uniform in the investigated period. When the testing policy changes, the value of the parameters $\alpha_{I_{S}H}$, α*_HT_*, and $\alpha_{{\text{HR}}^{d}}$ may vary. Here, we notice two important points. First, the value of $\alpha_{I_{S}T}$ is assumed to be constant in the whole period, as we suppose that there are no changes in how the symptomatic individuals requiring hospitalization are detected. Second, as a change in the sole parameter $\alpha_{I_{S}H}$ would affect too much the average time an individual remains infected, then α*_HT_* and $\alpha_{{\text{HR}}^{d}}$ also have to be included in the set of parameters that may change. On the basis of these considerations, the intervals in which each parameter remains constant or may change are identified. This defines the specific piece-wise waveform assumed for each of the parameters appearing in the model and, consequently, the effective number of values that need to be estimated for each parameter.

Hereafter, we summarize the events defining the different windows in which the whole period of investigation is partitioned:

1) On 02 March, a policy limiting screening only to symptomatic individuals is introduced.

2) On 12 March, a partial lockdown is issued.

3) On 18 March, a stricter lockdown, which further limits nonessential activities, is imposed.

4) On 29 March, a wider testing campaign is launched. Starting from this date, as the number of tests has constantly increased while the number of new infections was decreasing, the parameters are allowed to change every 14 or 28 days, namely, on 11 April, 25 April, and 23 May.

5) On 04 May, a partial lockdown lift is proclaimed.

6) On 18 May, further restrictions are relaxed.

7) On 03 June, interregional mobility is allowed. This is the last time the model parameters are changed.

Note that, for the time period until 5 April, we have followed the same time partition used in (*16*).

The model parameters have been estimated using a nonlinear optimization procedure (implemented via the function fmincon in MATLAB) with the following objective function to minimize

$$\begin{array}{l} e=\\ \sqrt{\frac{1}{4\tau }\sum_{k=1}^{\tau }({\left(H(k)-\bar{H}(k)\right)}^{2}+{\left(T(k)-\bar{T}(k)\right)}^{2}+{\left(R^{d}(k)-{\bar{R}}^{d}(k)\right)}^{2}+{\left(D(k)-\bar{D}(k)\right)}^{2})} \end{array}$$ (25)

where $\bar{H}(k)$, $\bar{T}(k)$, ${\bar{R}}^{d}(k)$, and $\bar{D}(k)$ with *k* = 1, …, τ (τ = 134 days) represent the time series of daily data for isolated, hospitalized, detected recovered, and deceased individuals provided by the Civil Protection Department (*49*), and *H*(*k*), *T*(*k*), *R*^d^(*k*), and *D*(*k*) are the values of the corresponding variables obtained from the integration of Eq. 24. The integration of Eq. 24 has been carried out by using a suitable ODE solver with maximum integration step size equal to 10^−2^ days and then resampling the data with a sampling period of 1 day.
